# Potential Use of Wollastonite as a Filler in UF Resin Based Medium-Density Fiberboard (MDF)

**DOI:** 10.3390/polym12071435

**Published:** 2020-06-27

**Authors:** Hamid R. Taghiyari, Ayoub Esmailpour, Roya Majidi, Jeffrey J. Morrell, Mohammad Mallaki, Holger Militz, Antonios N. Papadopoulos

**Affiliations:** 1 Wood Science and Technology Department, Faculty of Materials Engineering & New Technologies, Shahid Rajaee Teacher Training University, Tehran 16788-15811, Iran; mallaki.mammad@gmail.com; 2 Department of Physics, Faculty of Sciences, Shahid Rajaee Teacher Training University, Tehran 16788-15811, Iran; esmailpour@sru.ac.ir (A.E.); r.majidi@sru.ac.ir (R.M.); 3 National Centre for Timber Durability and Design Life, University of the Sunshine Coast, Brisbane, QLD 4102, Australia; jmorrell@usc.edu.au; 4 Wood Biology and Wood Products, Georg-August-University Göttingen, 37077 Göttingen, Germany; hmilitz@gwdg.de; 5 Laboratory of Wood Chemistry and Technology, Department of Forestry and Natural Environment, International Hellenic University, GR-661 00 Drama, Greece

**Keywords:** engineered materials, cell-wall polymers, wollastonite, wood-based composites

## Abstract

Urea-formaldehyde (UF) resins are primary petroleum-based, increasing their potential environmental footprint. Identifying additives to reduce the total amount of resin needed without adversely affecting the panel properties could reduce these impacts. Wollastonite is a mineral containing calcium and silica that has been used as an additive in a variety of materials and may be useful as a resin extender. Nanoscale wollastonite has been shown to enhance the panel properties but is costly. Micron-scale wollastonite may be a less costly alternative. Medium-density fiberboards were produced by blending a hardwood furnish with UF alone, micron-sized wollastonite alone, or a 9:1 ratio of UF to wollastonite. Panels containing of only wollastonite had poor properties, but the properties of panels with 9:1 UF/wollastonite were similar to the UF-alone panels, except for the internal bond strength. The results suggest that small amounts of micron-sized wollastonite could serve as a resin extender. Further studies are suggested to determine if the micron-sized material has similar positive effects on the resin curing rate.

## 1. Introduction

Wood composite panels are considered a growing field of products that are increasingly present in a variety of applications, with an undiminished upward trend now for many decades. It must be pointed out that one cannot speak about wood composites without speaking in depth about polymer binders and the adhesives used to hold them together. The history of wood composites is inextricably intertwined with the history and development of the polymer binders that are used to hold them together and manufacture them. In fact, not only has it been the continuous development of new or improved binders that has allowed the development of wood composites but, as presented later in this review, it is the continual renewal, discovery, and upgrading of such binders that has paved the way to further progress in the field of wood composites [1]. It must also be pointed out that the technique used to prepare a good wood composite is the mix of two very different technologies—namely, the use of a good adhesive, hence the capacity of formulating, engineering, and preparing one; and the technology of manufacturing the composite, this being particularly true in the case of wood panels. Thus, to obtain a good and acceptable wood composite, a balance between these two technologies is needed [1].

Urea-formaldehyde (UF) adhesives are by far the adhesive dominating the wood panel composites market for the preparation of interior-grade composite panels used for furniture and a wide variety of other applications. An approximate volume of 11 million tons/year of these adhesives for wood are used worldwide. Their technology has considerably progressed during the last few decades under the pressure of ever more stringent formaldehyde emission regulations. Notwithstanding their drawbacks, such as the lack of resistance to exterior weather conditions and the emission of formaldehyde, they are very difficult to be substituted for due to their relatively low cost, their excellent adhesive performance, and their ease of handling.

There are also several bio-based adhesives based on renewable natural materials that are at the forefront of new developments. Some of these are already used in industrial applications, such as tannin adhesives and some soy adhesives which have been using for many years, while others are on the threshold to industrialization, and many others are as yet at the experimental stage. The term “bio-based adhesive” has come to be used in a very well specified and narrow sense to only include those materials of natural, non-mineral origin which can be used as such or after small modifications to reproduce the behavior and performance of synthetic resins. Thus, only a limited number of materials can be currently included, at a stretch, in the narrowest sense of this definition [1]. These include tannins, lignin, carbohydrates, unsaturated oils, proteins, protein hydrolysates, dissolved wood, and wood welding by self-adhesion. Moreover, the environmental concern that predominates today has led to a considerable increase in research into and publication of bio-based adhesives for wood-based composites; therefore, the recent research on synthetic adhesives has made huge progress, with a host of new ideas and approaches having been thoughtout and presented. There are two distinct trends in this context: (i) the ongoing improvement of traditional synthetic adhesives, especially UF resins and melamine urea formaldehyde (MUF) resins, with valuable new ideas being presented [2,3]; and (ii) the presentation of partially non-traditional or even totally non-traditional synthetic adhesives, some also with some biobased content.

For the first type of approach, the tendency has been to prepare engineered UF resins of progressively lower molar ratios, as low as much less than 1:1 [4], this having become rather common in industry today. This was done with the aim to minimize the formaldehyde emission from wood panels bonded with UF resins. In the same trend, the potential introduction of a very acid pH condensation step in the preparation of UF resins inducing the formation of sometime considerable amounts of uron (a cyclic intramolecular urea methylene ether) in UF resins witha lower formaldehyde emission has caused some research interest [1,2]. Of interest is also the upgrading of UF, MUF, and PF resins by the addition of polymeric 4,4′ diphenyl methane diisocyanate (PMDI) directly to the water-based formaldehyde-based resin glue mix before application [5,6]. This was shown to lead to cross-linking by formation of traditional methylene bridges which simultaneously accompanied with formation of urethane bridges [5,6]. This is now a currently used industrial adhesive system, especially for UF and MUF resins, and was proved at the time to be the only system to prepare polyurethane bridges in water. Very recently, the catalytic influence of TiO_2_ in accelerating both the synthesis and cure of MF resins was found [1]. The effect is noticeable and could be due to a variety of causes. For non-traditional synthetic adhesives, the first shocking concept was that urea-formaldehyde can be classed as a bio-adhesive too. Urea is considered bio-derived from the nitrogen in the air; but while formaldehyde exists in nature, its industrial production is not bio at all. Thus, the interest is growing in eliminating formaldehyde with something less or not at all toxic and especially non-volatile (to eliminate formaldehyde emission). The first attempts to solve this problem led to the preparation of urea-glyoxal (UG) resins for wood composite adhesives [7,8]. Urea-glyoxal resins are already well-developed and used in the textile industry, but the formulations needed to be extensively changed for wood-based composites. The approach progressed to IL (ionic liquids as hardeners), MG (melamine-glyoxal), and finally to IL melamine-glyoxal-glutaraldehyde (IL MGG’) adhesives, which gave a very acceptable performance as adhesives for wood composites [9].

Many minerals have been added to resins as reinforcing fillers to improve the composite properties [10,11,12,13,14,15,16,17,18]. Silicate minerals have been shown to enhance the thermal conductivity in oriented strandboard, thereby reducing the potential energy [19,20,21,22]. Nanoscale wollastonite has also been shown to improve the physical and mechanical properties of medium-density fibreboard and particleboard, possibly via bonding between wollastonite and wood cell-wall polymers [23,24]. Nano-wollastonite has been shown to act as a reinforcing filler to improve the shear strength of polyvinyl acetate resin, as a fire-retardant additive for wood coatings, or as an additive for the production ofMedium-Density Fiberboard(MDF) or particleboard panels [23,25,26,27,28]. While nano-wollastonite has many attractive properties, its high cost has limited its commercial application. The objective of the current study was to investigate the potential for using micron-scale wollastonite alone or as an additive/extender in UF resin for manufacturing medium-density fibreboard with acceptable properties. It is to be noted that micron-scale wollastonite has a competitive price, and in case some improvements are achieved in the properties of the produced MDF panels, the industry sector will be interested in its application at an industrial scale.

## 2. Materials and Methods

### 2.1. Panel Production

#### 2.1.1. Materials

A mixture of dried fibres consisting mostly of beech (*Fagus orientalis*), alder (*Alnusglutinosa*), hornbeam (*Carpinusbetulus*), and poplar (mostly *Populusnigra)* along with approximately 5% pruned branches from miscellaneous species was obtained from Sanaye Choobe Khazar Caspian Company (Amol, Iran). UF resin (1.277 g/cm^3^) was obtained from Pars Chemical Industries Co (Tehran, Iran). The viscosity and gel time of pure UF resin and UF resin with 10% wollastonite (9:1 UF/wollastonite ratio) are shown in Table 1. Micron-sized wollastonite gel (55% solids) was purchased from Mehrabadi Manufacturing Company (Tehran, Iran). The price of micron-sized wollastonite is not very high in comparison to UF resin, and therefore its application would be quite economical in case some improvements in the properties of composite panels are achieved.

#### 2.1.2. Panel Production

The wood fibers were blended in a rotary drum while urea formaldehyde alone, wollastonite alone, or a 9:1 UF/wollastonite mixture was sprayed onto the fibers at a ratio of 10% resin/additive and 90% wood fibers. The ratio 9:1 UF/wollastonite was chosen based on previous studies in which 10% of wollastonite was reported to be the optimum level [17,23]. The materials were formed into mats that were pressed for 4 min at 170 °C and 160 bars to a target thickness of 16 mm and a panel density of 0.67 g/cm^3^. The panels were then conditioned at 25 ± 3 °C and 42 ± 3% relative humidity for two weeks before being cut to size. The panel moisture content, as determined by oven drying small samples, was 7.5% at the time of testing. Three replicate panels were produced for each treatment. The outer 50 mm was trimmed from each panel, then samples measuring 50 by 50 mm (water absorption/thickness swell and internal bond), 75 by 75 mm (for screw holding capacity), and 50 by 350 mm long (for flexural testing) by panel thickness were cut. Additional samples (200 by 150 mm by panel thickness) were cut for assessing the fire resistance.

### 2.2. Physical and Mechanical Tests

Physical and mechanical tests, as well as recording the number and location of the specimens, were carried out in accordance with the Iranian National Standard ISIRI 9044 PB Type P2 (Iranian National Standards Organization, Tehran, Iran), compatible with the ASTM [29]. All the tests were performed on an Instron 4486 universal testing machine (Norwood, MA, USA).

#### 2.2.1. Water Absorption and Thickness Swell

The samples were weighed and their dimensions measured with digital calipers (nearest 0.01 mm) before being immersed in distilled water. The differences in weight and dimensions were measured after 2 and 24 h of immersion and changes were used to calculate the % water absorption and thickness swell. Each treatment was replicated on 6 samples.

#### 2.2.2. Internal Bond Strength

The wide faces of the 50 by 50 mm samples were glued to slotted aluminum blocks that were then pulled apart on an Instron 4486 universal testing machine, and the load required to achieve separation was recorded. Six samples were tested for each panel type.

#### 2.2.3. Flexural Tests

The 50 by 350 mm-long beams were tested in third-point loading at a span of 320 mm at a loading rate of 3 mm/min. The load and deflection were continuously recorded and the resulting data were used to calculate the modulus of rupture (MOR) and modulus of elasticity (MOE). Each material was tested on 3 samples.

#### 2.2.4. Screw Withdrawal Test

Screw withdrawal tests were performed on the faces and edges of 75 mm square sections using 4.25 mm-diameter MDF screws (Sanaye Pich Iran, Tehran, Iran) in accordance with ASTM standard D 1761-88 at a withdrawal speed of 2.5 mm/min [30]. A 2 mm pilot hole was drilled prior to inserting the screws to a depth of 17 mm in the panels, leaving 1 mm of the screw above the panel surface for testing. Six replicate specimens were tested for each panel type. The specimens were conditioned at 25 ± 3 °C and 42 ± 3% relative humidity for a month before testing.

#### 2.2.5. Fire Resistance

The fire resistance was measured on a modified device patterned after the system described in ISO Standard 119525-3 [26,27]. The system consisted of a Bunsen burner on a sliding stand at 45 °C to the panel set so that the burner could slide towards and away from the panel. The flame was provided by a methane-based natural gas at a flow rate of 5.8 L/min. The specimen was exposed to the flame for 120 s and the time required for the onset of ignition (i.e., visible flame) as well as the time required for the panel to glow (time of glowing onset) were recorded. The burner was withdrawn and the time that visible flame remained was recorded. After cooling, the burned area was measured.

### 2.3. Modelling by Density Functional Theory (DFT) 

Simulations were used to examine how wollastonite might interact with carbohydrates in wood cell-wall polymers using OpenMX3.8 (Tokyo, Japan) through density functional theory (DFT) [31,32,33,34,35]. Possible interactions were simulated using generalized gradient approximation (GGA), as described by Perdew–Burke–Ernzerhof (PBE) [36]. Long-range van der Waals interactions were included in the simulations by the DFT-D2 approach. The cutoff energy in the plane wave was set to 50 Ry in a uniform way and in all three cell-wall components. The basis set of s1p1d1 pseudo-atomic orbit with a cutoff distance of 7 bohr radii and fully relativistic pseudopotentials (PBE13) was taken from the 2013 database [37,38].

The potential for interactions between wollastonite and the cell-wall polymers were calculated using Equation (1).

*E*_ads_ = *E*_cellulose/hemicellulose/lignin + W_ − (*E*_cellulose/hemicellulose/lignin_ + *E*_W_), (1)

where *E*_cellulose/hemicellulose/lignin + W_ is the total energy of cellulose, hemicellulose, or lignin with adsorbed W; *E*_cellulose/hemicellulose/lignin_ represents the total energy of the isolated polymers (existing in the cellwall); and *E*_W_ is the total energy of the isolated wollastonite. In this evaluation, the negative adsorption energy was translated into a more stable adsorption structure. The details of the total energy calculation in DFT are presented in the Supplementary Materials (SM) 1. 

**Figure 1:**
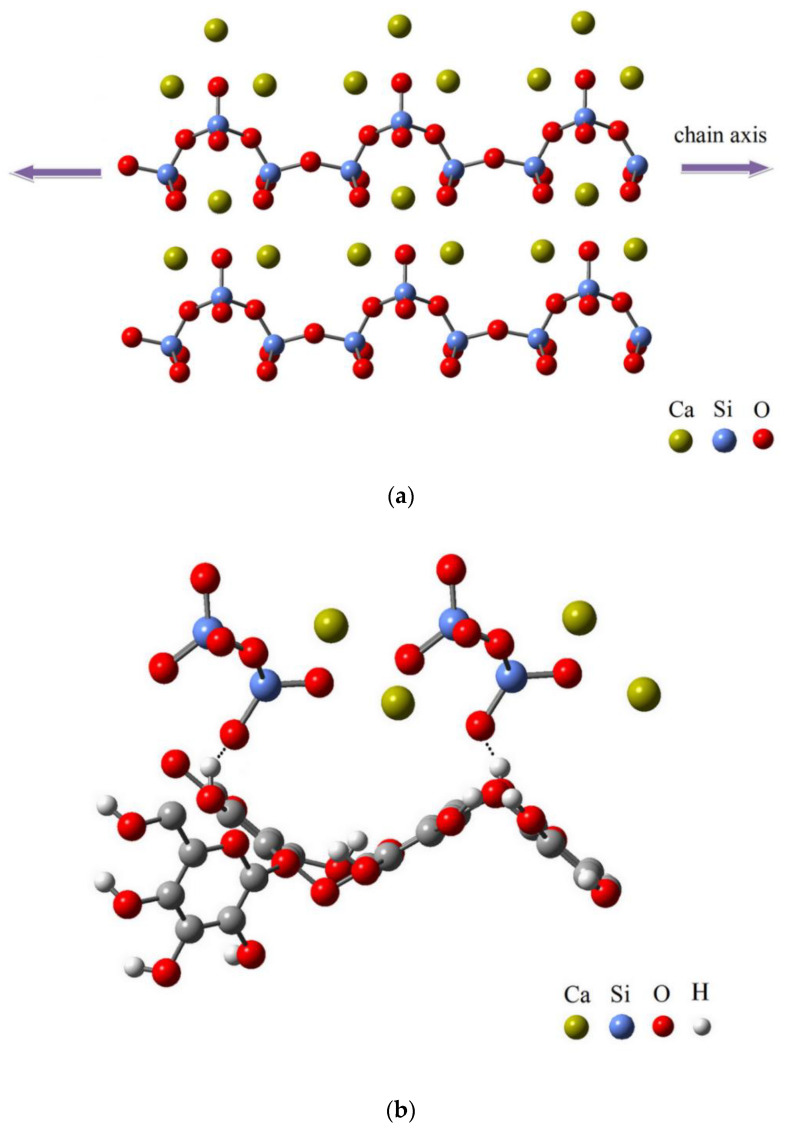

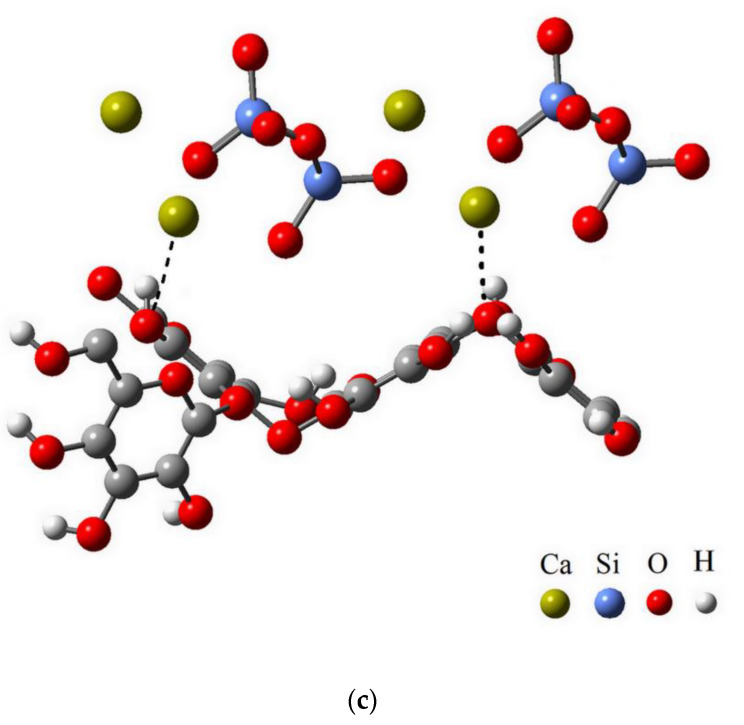
Predicted atomic structures of: wollastonite (**a**), side view of two models of the most stable configurations of wollastonite adsorbed on hemicellulose, showing bonds between hydroxyl group of hemicellulose with oxygen atom (**b**) and Ca (**c**) of wollastonite.

The silicate chains along the axis of the wollastonite crystals (Figure 1a) are linked to a periodicity of three tetrahedra. Calcium molecules are linked to six of the oxygen molecules by octahedral coordination [39,40,41].

The model originally introduced by Kaith et al. and Pizzi [42,43,44] was used to measure and calculate the potential adsorption of wollastonite as well as any water molecules on hemicellulose.

### 2.4. Statistical Analysis of Data

The data from the physical property measurements were analyzed using a one-way analysis of variance (ANOVA) (α = 0.05) using the SAS software (version 9.2, 2010). Differences between the individual treatments were analyzed using Duncan’s multiple range test using SPSS/18 (2010).

## 3. Results

### 3.1. DFT Modelling of Adsorption of Wollastonite

The wollastonite molecule was rotated on the surface of each cell-wall polymer and moved closer together to identify the optimal adsorption distance and minimum adsorption energy. The configuration with the minimum adsorption energy was considered as the most stable configuration. Two models of the most stable configurations of wollastonite adsorbed on hemicellulose are shown in Figure 1b. The nearest distance between the wollastonite and hemicellulose surface and the adsorption energy of the most stable structure were 1.7 Å and −4.5 eV, respectively (Figure 1b). The hydrogen bonds formed between the hydroxyl group of hemicellulose and the oxygen atom of wollastonite (OH_hemicellulose_∙∙∙O_W_) (Figure 1b). The bonds between Ca in the wollastonite and hydroxyl groups (Ca∙∙∙OH_hemicellulose_) and the wollastonite and epoxy groups (Ca∙∙∙O_hemicellulose_) of hemicellulose (Figure 1c) were instrumental in the adsorption of wollastonite on the hemicellulose molecules. The adsorption distance between the wollastonite and hydroxyl as well as the epoxy groups was 2.50 Å (the dot lines in Figure 1c and Table 2).

The wollastonite adsorption energy on cellulose was stronger than that on hemicellulose (Table 2), possibly because the branching on the hemicellulose interfered with the interactions. 

The DFT configuration of the water molecules on hemicellulose revealed that the adsorption energy increased with increasing numbers of water molecules (0.85 to 9.34 eV, from 1 to 12 molecules, respectively) (Figure 1). This increase in turn decreased the likelihood that the wollastonite would be adsorbed on the hemicellulose.

The most stable calculated wollastonite configuration on lignin had average adsorption distances and energies of 1.8 Å and −2.6 eV, respectively (Table 2). The low adsorption energy of wollastonite molecules on lignin provided little evidence of substantial interactions. Positive adsorption energies of water molecules on coumaryl, coniferyl and sinapyl alcohols are consistent with previous studies indicating that lignin is a hydrophobic polymer (Table 2) [45,46,47,48,49,50].

The interaction between the wollastonite and UF resin was also studied. Though not very strong, the adsorption energy of −2.7 eV indicates the formation of a binding between the wollastonite and UF resin.

### 3.2. Mechanical Properties

The Modulus of Rupture (MOR): MOR values for the conventional UF resin panels averaged 11.2 MPa. Panels with 9:1 UF/wollastonite demonstrated a 6% reduction in MOR; however, this reduction was not statistically significant as they were both in the same Duncan’s grouping (Table 3). The decrease was surprising, since previous studies indicated that nano-wollastonite addition to UF resin improved panel properties [23]. The properties of UF resin (Table 1) indicate that the addition of wollastonite to the UF resin had no significant effect on the pH and viscosity of the resin. Wollastonite increased the gel time; however, the gel time of 9:1 UF/wollastonite is still well below the hot-press time of four minutes. Therefore, it can be assumed that the alteration in gel time had little effect on the properties. In this connection, further studies on the effects of the addition of wollastonite to UF resin should be done with a wide range of ratios (including 10:0.5, 9.5:0.5, 9:1, 8.5:1.5, 8:2) to come to a final conclusion in this regard. The slight reduction in the present study could be attributed to the slightly lower amount of UF. In the previous studies, nano-wollastonite was added to UF resin with no reduction in the total UF content. Wollastonite has been shown to provide reinforcement in different resins (UF and polyvinyl acetate) and paints [19,23,25,27]. The substitution of wollastonite for all of the UF resin resulted in a more than 80% loss in MOR compared with the UF control. The results suggest that wollastonite has minimal interactions with wood fibers, resulting in poor adhesion characteristics. These results are supported by the modeling studies showing little interaction between wollastonite and cellulose, hemicellulose, or lignin. 

Panels with 9:1 UF/wollastonite content showed a non-significant difference in MOE compared to the control panels (UF resin), suggesting that the effects on this property might be proportional to the additive level. Substituting wollastonite for all of the UF resin was associated with a 75% decrease in the panel MOE. 

Panels with 9:1 UF/wollastonite also experienced a significant loss (37%) in internal bond (IB) strength, while the complete substitution of wollastonite for UFresin was associated with a nearly 70% loss in IB. Previous studies have shown that nano-wollastonite caused small but insignificant reductions in IB [23]. IB is a measure of the degree to which the adhesive interacts with the wood fibres, and it is clear that the addition of wollastonite has a disproportionate effect on this property.

The partial substitution of wollastonite for UF resin (9:1 UF/wollastonite panels) was associated with no statistically significant difference in both the edge and face fastener withdrawal compared to the UF control panels, representing 11% and 7% increases in this property, respectively. The complete substitution of wollastonite for UF resin resulted in 51% and 70% reductions in the fastener withdrawal resistance from the face and edge, respectively. Fastener resistance is a function of fastener design, moisture content, and material density. Neither the fastener design nor moisture content differed in these tests; however, the sharp decrease in withdrawal resistance suggests that the fiber/adhesive interactions had decreased. These results would be consistent with declines in the MOR, MOE, and IB and indicate that the wollastonite had minimal interactions with the wood fibres, leading to the reduced properties.

### 3.3. Physical Properties

Panels with a 9:1 UF/wollastonite content showed no significant difference in water absorption (WA) or thickness swelling (TS) after 2 and 24 h of immersion in water (Table 4). Nano-wollastonite had previously been shown to improve these properties; however, wollastonite in that study was added to the total 10% UF resin, suggesting that the substitution of wollastonite for 10% UFresin in the current tests may have offset the effects of the lower resin content [23]. The water absorption in the panels made using only wollastonite was nearly three times higher than that found in the panels made with UF resin alone after 2 h of immersion and 2.7 times higher after 24 h. The thickness swelling followed similar trends in the panels, with the ones with UF alone and a 9:1 UF/wollastonite content having similar thickness changes, while the panels composed of fibers plus only wollastonite experienced swelling levels that were nearly 5 and 4 times higher than those for the UF panels after 2 and 24 h of immersion, respectively. The results indicate that some addition of wollastonite had no significant effect on the swelling or water absorption, but eliminating all the UF resin produced panels with unacceptable properties.

### 3.4. Fire Properties

The substitution of wollastonite for 10% UF resin (9:1 UF/wollastonite panels) was associated with a non-significantly reduced time to the onset of ignition (5% faster) but a slower time to the onset of glowing (15%) (Table 5). These results suggest that wollastonite acted as an initial accelerant that would normally lead to the formation of a char layer that inhibited further oxygen movement. The burn duration and total area burned for the 9:1 panels were similar to those with only UF resin, suggesting that the addition of a small amount of wollastonite had no significant effect on the fire properties.

The production of panels where wollastonite was substituted for all of the UF resin resulted in very different fire properties. The time to the onset of ignition was almost 40% faster in the wollastonite panels, although the time to the onset of glowing was the same. The burn duration was 25% shorter for the wollastonite panels, but the total burned area was almost 3.5 times greater. The results support the premise that wollastonite functions to enhance ignition over a broader panel surface, but the resulting char layer reduced the burn time. These results would be consistent with previous studies showing that wollastonite could act as a panel fire retardant, but these potential benefits were heavily offset by the reduced mechanical properties and increases in the thickness swelling and water absorption.

## 4. Conclusions

The objective of the current study was to examine the potential of using micron-scale wollastonite alone or as an additive/extender in UF resin for manufacturing medium-density fiberboard. Panels were produced by blending a hardwood furnish with UF alone, micron-sized wollastonite alone, or a 9:1 ratio of UF to wollastonite. The panels containing only wollastonite had poor properties, but the properties of panels with 9:1 UF/wollastonite were similar to the UF-alone panels except for the internal bond strength. With regard to the economical price of micron-sized wollastonite in comparison to UF resin, the results suggest that small amounts of micron-sized wollastonite could serve as a resin extender. Further studies are suggested to determine if the micron-sized material has similar positive effects on the resin curing rate. Moreover, the possible effect of the addition of this mineral on machining equipment should be investigated in a separate study.

## Figures and Tables

**Table 1:** Viscosity, pH, and gel time of urea formaldehyde (UF) resin and UF resin with 10% wollastonite.

Amount Added (%) ^1^		Average Value ^2^			
UF Resin	Wollastonite	Gel Time (s)	pH	Viscosity (cP)	
				6 rpm	10 rpm
10	0	65	7.25	450	455
9	1	90	6.95	430	435

^1^ Total amount of material added to the wood was 10 % *w*/*w*. ^2^ Values represent the averages of 3 replicates.

**Table 2:** Calculated adsorption energies and the nearest adsorption distances of wollastonite molecules on the surfaces of cellulose, hemicellulose, and lignin molecules (O and OH represent the epoxy and hydroxyl groups of cellulose, hemicellulose, and lignin, respectively).

Molecules	Adsorption Distance (Å)		Wollastonite Adsorption Energy(eV)	Adsorption Energy of One Water Molecule (eV)	Adsorption Energy of Abundant Water Molecules (eV)
	OH O_W_	CaOH CaO			
Cellulose ^1^	1.7	2.45	−6.6	−1.1	−14.8
Hemicellulose	1.7	2.50	−4.5	−0.85	−9.34
Lignin	1.8	2.51	−2.6	+	+

^1^ The values are reported from Taghiyari et al. [24].

**Table 3:** Effect of the addition of wollastonite on the mechanical properties of Medium-Density Fiberboard (MDF) panels.

Amount Added (%) ^1^		Average Value (MPa) ^2^				
UF Resin	Wollastonite	MOR	MOE	Internal Bond	Fastener Withdrawal	
					Face	Edge
10	0	11.2 (1.3) a	1023 (95) a	0.32 (0.02) a	650 (75) a	270 (30) a
9	1	10.5 (1.4) a	900 (100) a	0.2 (0.01) b	700 (68) a	300 (34) a
0	10	2 (0.15) b	250 (3) b	0.1 (0.01) c	320 (35) b	80 (10) b

^1^ Total amount of material added to the wood was 10 % *w*/*w*. ^2^ Values represent the averages of 3 replicates per modulus of rupture (MOR) and modulus of elasticity (MOE) tests and 6 replicates for internal bond (IB) and fastener withdrawal tests, while the figures in parentheses represent one standard deviation. Values followed by the same letter for a given property do not differ from one another by Duncan’s multiple range test (α = 0.05).

**Table 4:** Effect of the addition of wollastonite on the water absorption and thickness swelling of MDF panels.

Amount Added (%) ^1^		Water Absorption (%) ^2^		Thickness Swelling (%) ^2^	
UF Resin	Wollastonite	2 h	24 h	2 h	24 h
10	0	115 (10) b	163 (15) b	24 (2.3) b	35 (3) b
9	1	117 (10.5) b	177 (17) b	24 (2.5) b	33 (3.2) b
0	10	320 (20.5) a	434 (30.4) a	118 (12.5) a	138 (13.5) a

^1^ Total amount of material added to the wood was 10 % *w*/*w*. ^2^ Values represent the averages of 6 replicates per test, while the figures in parentheses represent one standard deviation. Values followed by the same letter for a given property do not differ from one another by Duncan’s multiple range test (α = 0.05).

**Table 5:** Effect of the addition of micron wollastonite on the fire performance of MDF panels subjected to a flame exposure test. ^1^

UF Resin (%)	Wollastonite (%)	Total Time (s) ^2^			Burn Area (mm^2^) ^2^
		To Onset of Ignition	To Onset of Glow	Burn Time	
10	0	18.9 (1.5) a	46 (4) b	1.2 (0.09) a	2878 (200) b
9	1	18 (1.7) a	53 (4.5) a	1.2 (0.1) a	2851 (230) b
0	10	11.2 (1.6) b	45 (4.5) b	0.9 (0.1) b	10093 (950) a

^1^ Total amount of material added to the wood was 10 % *w*/*w*. ^2^ Values represent the averages of 3 replicates per test, while the figures in parentheses represent one standard deviation. Values followed by the same letter for a given property do not differ from one another by Duncan’s multiple range test (α = 0.05).

## Supplementary Materials

The following are available online at https://www.mdpi.com/2073-4360/12/7/1435/s1, Details of the total energy calculation in DFT.
