# Supplementary material for: Potential Use of Wollastonite as a Filler in UF Resin Based Medium-Density Fiberboard (MDF)
Source: Polymers (Basel). 2020 Jun 27;12(7):1435. doi: 10.3390/polym12071435 (PMC7408164; doi:10.3390/polym12071435)
Supplement: Supplementary file 1 [file polymers-12-01435-s001.pdf]

polymers

MDPI

SUPPLEMENTARY MATERIAL 1

Details of the total energy calculation in DFT.

The total energy, E_{tot}, in density functional theory (DFT) is given by the sum of the kinetic energy, E_{kin}; the electron-core Coulomb energy, E_{ec}; the electron-electron Coulomb energy, E_{ee}; the exchange-correlation energy, E_{xc}; and the core-core Coulomb energy, E_{cc}, between the pseudocore charges Z_i and Z_j as follows:

$$E_{tot} = E_{kin} + E_{ec} + E_{ee} + E_{xc} + E_{cc}, \quad (1)$$

with

$$E_{kin} = 2\sum_{i\alpha,j\beta} \rho_{i\alpha,j\beta} \int d(r)\chi_{i\alpha}\hat{T}\chi_{i\beta}, \quad (2)$$

$$E_{ec} = E_{ec}^{(L)} + E_{ec}^{(NL)} = \int d(r)n(r)\sum_k V_{L,k}(r-R_k) + \int d(r)n(r)\sum_k NV_{L,k}(r-R_k), \quad (3)$$

$$E_{ee} = \frac{1}{2}\int\int d(r)d(r')\frac{n(r)n(r')}{|r-r'|}, \quad (4)$$

$$E_{xc} = \int d(r)n\varepsilon_{xc}(n), \quad (5)$$

$$E_{cc} = \frac{1}{2}\sum_{i,j}\frac{Z_i Z_j}{|R_i - R_j|}, \quad (6)$$

where i and α are the site and basis function indices, respectively. $\rho_{i\alpha,j\beta}$ is the density matrix associated with two basis functions, i and j, and is defined by $\chi_{i\alpha}$ and $\chi_{j\beta}$. These are defined by $\sum_v \Theta(\varepsilon_v - \mu)c_{i\alpha,v}c_{j\beta,v}$, with the linear combination of numerical atomic local basis orbitals (LCAO) coefficient, $c_{i\alpha,v}$; the one particle eigen-energy, ε_v; the chemical potential, μ; and a step function, $\Theta(x)$. n is the electro density defined by $2\sum_{i\alpha,j\beta}\rho_{i\alpha,j\beta}\chi_{i\alpha}\chi_{j\beta}$. $V_{L,k}$ and $V_{NL,k}$ are the local and non-local parts in the norm-conserving pseudopotential of atom k, respectively. The factor 2 in E_{kin} and n is for the spin multiplicity [1].

[1] Ozaki, T., Kino, H., Efficient projector expansion for the ab initio LCAO method. *Physical Review* B 2005, 72, 045121.
